# Supplementary figures and images for: Microbiota responses to different prebiotics are conserved within individuals and associated with habitual fiber intake
Source: Microbiome. 2022 Jul 29;10:114. doi: 10.1186/s40168-022-01307-x (PMC9336045; doi:10.1186/s40168-022-01307-x)

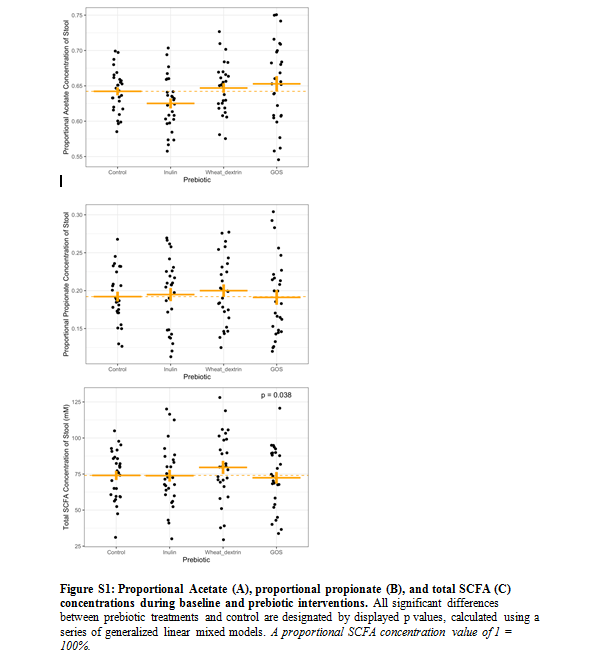

Supplement: Supplementary file 2 — Additional file 1. (A), proportional propionate (B), and total SCFA (C) concentrations during baseline and prebiotic interventions. All significant differences between prebiotic treatments and control are designated by displayed p values, calculated using a series of generalized linear mixed models. A proportional SCFA concentration value of 1 = 100%. [file 40168_2022_1307_MOESM1_ESM.png]

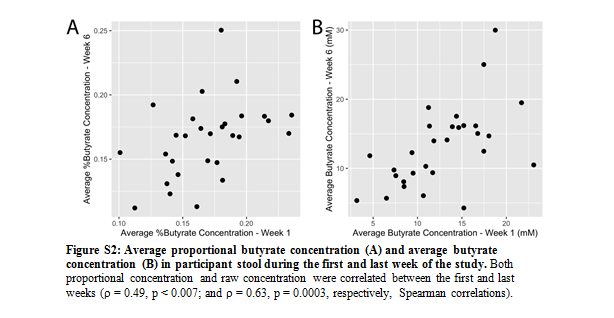

Supplement: Supplementary file 3 — Additional file 2. Average proportional butyrate concentration (A) and average butyrate concentration (B) in participant stool during the first and last week of the study. Both proportional concentration and raw concentration were correlated between the first and last weeks (ρ = 0.49, p < 0.007; and ρ = 0.63, p = 0.0003, respectively, Spearman correlations). [file 40168_2022_1307_MOESM2_ESM.png]

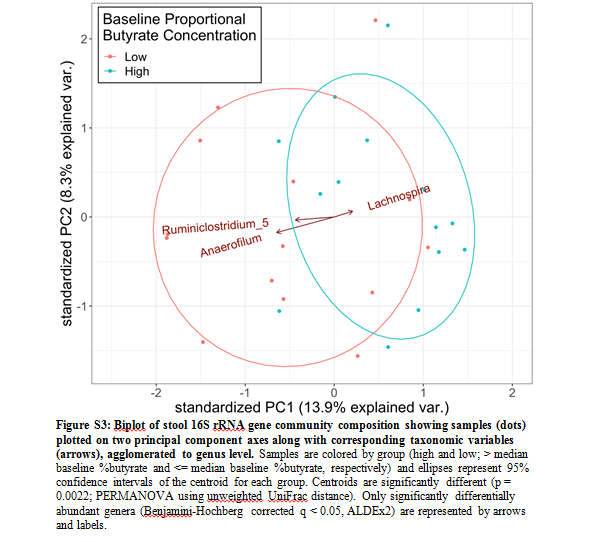

Supplement: Supplementary file 4 — Additional file 3. Biplot of stool 16S rRNA gene community composition showing samples (dots) plotted on two principal component axes along with corresponding taxonomic variables (arrows), agglomerated to genus level. Samples are colored by group (high and low; > median baseline %butyrate and <= median baseline %butyrate, respectively) and ellipses represent 95% confidence intervals of the centroid for each group. Centroids are significantly different (p = 0.0022; PERMANOVA using unweighted UniFrac distance). Only significantly differentially abundant genera (Benjamini-Hochberg corrected q < 0.05, ALDEx2) are represented by arrows and labels. [file 40168_2022_1307_MOESM3_ESM.png]

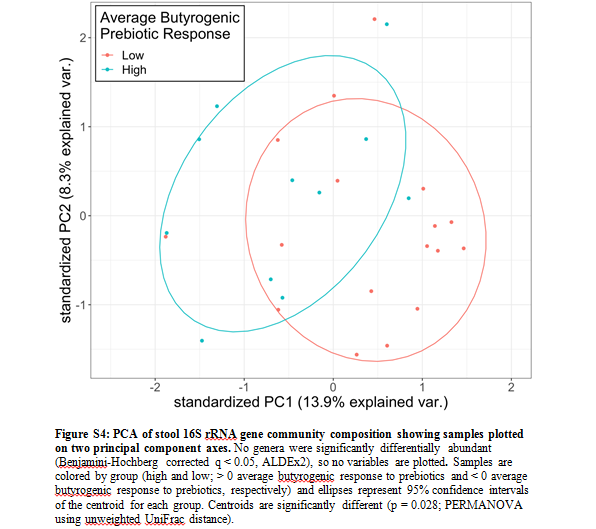

Supplement: Supplementary file 5 — Additional file 4. PCA of stool 16S rRNA gene community composition showing samples plotted on two principal component axes. No genera were significantly differentially abundant (Benjamini-Hochberg corrected q < 0.05, ALDEx2), so no variables are plotted. Samples are colored by group (high and low; > 0 average butyrogenic response to prebiotics and < 0 average butyrogenic response to prebiotics, respectively) and ellipses represent 95% confidence intervals of the centroid for each group. Centroids are significantly different (p = 0.028; PERMANOVA using unweighted UniFrac distance). [file 40168_2022_1307_MOESM4_ESM.png]

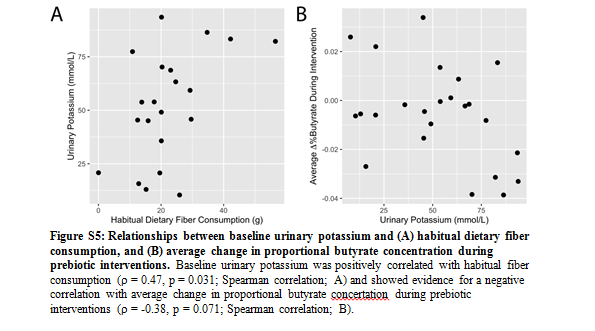

Supplement: Supplementary file 6 — Additional file 5. Relationships between baseline urinary potassium and (A) habitual dietary fiber consumption, and (B) average change in proportional butyrate concentration during prebiotic interventions. Baseline urinary potassium was positively correlated with habitual fiber consumption (ρ = 0.47, p = 0.031; Spearman correlation; A) and showed evidence for a negative correlation with average change in proportional butyrate concentration during prebiotic interventions (ρ = -0.38, p = 0.071; Spearman correlation; B). [file 40168_2022_1307_MOESM5_ESM.png]

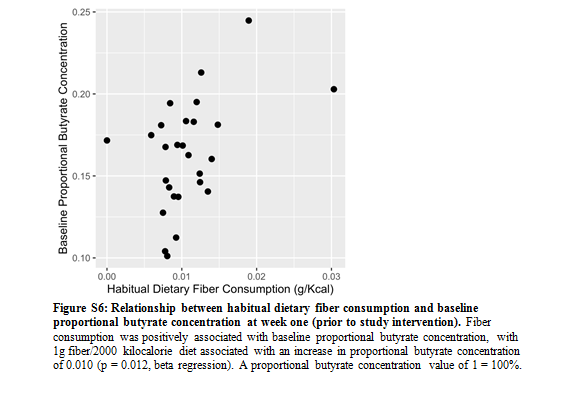

Supplement: Supplementary file 7 — Additional file 6. Relationship between habitual dietary fiber consumption and baseline proportional butyrate concentration at week one (prior to study intervention). Fiber consumption was positively associated with baseline proportional butyrate concentration, with 1g fiber/2000 kilocalorie diet associated with an increase in proportional butyrate concentration of 0.010 (p = 0.012, beta regression). A proportional butyrate concentration value of 1 = 100%. [file 40168_2022_1307_MOESM6_ESM.png]

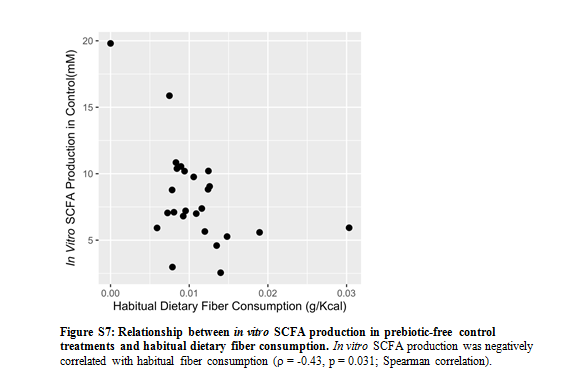

Supplement: Supplementary file 8 — Additional file 7. Relationship between in vitro SCFA production in prebiotic-free control treatments and habitual dietary fiber consumption. In vitro SCFA production was negatively correlated with habitual fiber consumption (ρ = -0.43, p = 0.031; Spearman correlation). [file 40168_2022_1307_MOESM7_ESM.png]
